# The complete chloroplast genome of wild *Magnolia officinalis* Rehd. et Wils

**DOI:** 10.1080/23802359.2021.1886011

**Published:** 2021-03-15

**Authors:** Shanyong Yi, Xiangwen Song, Wangyang Yu, Wei Wang, Tao Xu, Rongfei Zhang, Dong Liu, Bangxing Han

**Affiliations:** a Department of Biological and Pharmaceutical Engineering, West Anhui University, Luʼan, P.R. China; b Anhui Engineering Laboratory for Conservation and Sustainable Utilization of Traditional Chinese Medicine Resources, West Anhui University, Luʼan, P.R. China; c Research and Development Department, Anhui Qiansouyan Biotechnology Co., Ltd, Luʼan, P.R. China; d State Key Laboratory of Natural Medicines, Department of TCMs Pharmaceuticals, School of Traditional Chinese Pharmacy, China Pharmaceutical University, Nanjing, P.R. China

**Keywords:** *M. officinalis*, complete chloroplast genome, phylogenetic analysis

## Abstract

*Magnolia officinalis* Rehd. et Wils. is an important traditional Chinese herbal medicine and widely distributed in the south of Yangtze River in China. In this study, the complete chloroplast genome sequence of wild *M. officinalis* was assembled and characterized from high-throughput sequencing data. The chloroplast genome was 160,009 bp in length, consisting of large single-copy (LSC) and small single-copy (SSC) regions of 88,134 bp and 18,739 bp, respectively, which were separated by a pair of 26,568 bp inverted repeat (IR) regions. The genome is predicted to contain 132 genes, including 87 protein-coding genes, 37 tRNA genes, and 8 rRNA genes. The overall GC content of the genome is 39.3%. A phylogenetic tree reconstructed by 86 chloroplast genomes reveals that *M. officinalis* is mostly related to cultivated *M. officinalis, M. obovata* and *M. tripetala.*

*M．officinalis* is a deciduous tree belonging to the *Magnolia* genus of Magnoliaceae (Xia et al. 2010). As one of the three woody medicinal materials in China, it has been commonly used in the treatment of eliminating dampness and dissolving phlegm more than 2000 years (Zhang 2005). Pharmacological analysis showed that *M. officinalis* has the ability of anti-tumor (Youn et al. 2013), anti-bacterial (Liu et al. 2014), anti-anxiety (Han et al. 2011), and liver protection (Xie et al. 2015). According to statistics, there are more than 200 kinds of Chinese patent medicines containing *M. officinalis*, and the annual demand is more than 6000 tons (Yang et al. 2017). Causing on the dried root bark, trunk bark and branch bark of *M. officinalis* are used as medicine, it is usually harvested by peeling after felling at the cost of the whole plant death. Driven by market interests, there were two large-scale felling in China in the 1960s and 1980s, which led to a sharp decline in the number of wild resources of *M. officinalis*. Now *M. officinalis* is an endangered Chinese medical plant (Yang et al. 2018) and listed as the second-class protected plant in China (Fu 1992). And the results of field investigation showed that the wild *M. officinalis* was hard to find based on a large number of poaching. So the populations have been dramatically decreased in recent decades and urgently need protection and restoration, especially the wild *M. officinalis*. Despite the chloroplast (cp) genome of cultivated *M. officinalis* has been reported (Li et al. 2012), little is known about the cp genome of wild *M. officinalis*. And, due to the large number of SNPs and about 180 bp indel, the cp genome of *M. officinalis* reported in this study is different from the cp genome of *M. officinalis* previously reported, which indicates that *M. officinalis* may have a high genetic diversity. Here, the complete cp genome of wild *M. officinalis* using high throughput sequencing technology was determined, which will provide informatics data for the phylogeny of *Magnolia* genus and further enrich the evolutionary research on *M. officinalis*.

The fresh leaves of wild *M. officinalis* were sampled from Lu’an, Anhui, China (31°77′ N, 115°93′ E). Specimens were stored in the Herbarium of West Anhui University (accession number: WAU-HP-20201018-1). Total genomic DNA was extracted with a modified CTAB protocol (Doyle and Doyle 1987). The whole genome sequencing was conducted by Hefei Biodata Biotechnologies Inc. (Hefei, China) on the Illumina Hiseq platform. The filtered sequences were assembled using the program SPAdes assembler 3.10.0 (Bankevich et al. 2012). Annotation was performed using the DOGMA (Wyman et al. 2004) and BLAST searches.

The cp genome of *M. officinalis* was determined to comprise a 160,009 bp double stranded, circular DNA (Genome accession no. MW373503/GWHAOSH01000000), which containing two inverted repeat (IR) regions of 26,568 bp, separated by large single-copy (LSC) and small single-copy (SSC) regions of 88,134 bp and 18,739 bp, respectively. The overall GC content of *M. officinalis* cp genome is 39.3% and the corresponding values in LSC, SSC and IR regions are 38.0%, 34.3% and 43.2%, respectively. The cp genome was predicted to contain 132 genes, including 87 protein-coding genes, 37 tRNA genes, and 8 rRNA genes. Seven protein-coding genes, seven tRNA genes and four rRNA genes were duplicated in IR regions. Seventeen genes contained two exons and four genes (*clpP, ycf3* and two *rps12*) contained the exons.

To investigate its taxonomic status, alignment was performed on the 86 cp genome sequences using MAFFT v7.307 (Katoh and Standley 2013), and a maximum likelihood (ML) tree was constructed by FastTree version 2.1.10 (Price 2010). As expected, *M. officinalis* is mostly related to cultivated *M. officinalis, M. obovata* and *M. tripetala* with bootstrap support values of 100% (Figure 1). The complete cp genome sequence of *M. officinalis* will provide a useful resource for the conservation genetics of this species as well as for the phylogenetic studies of *Magnolia*.

**Figure 1.**
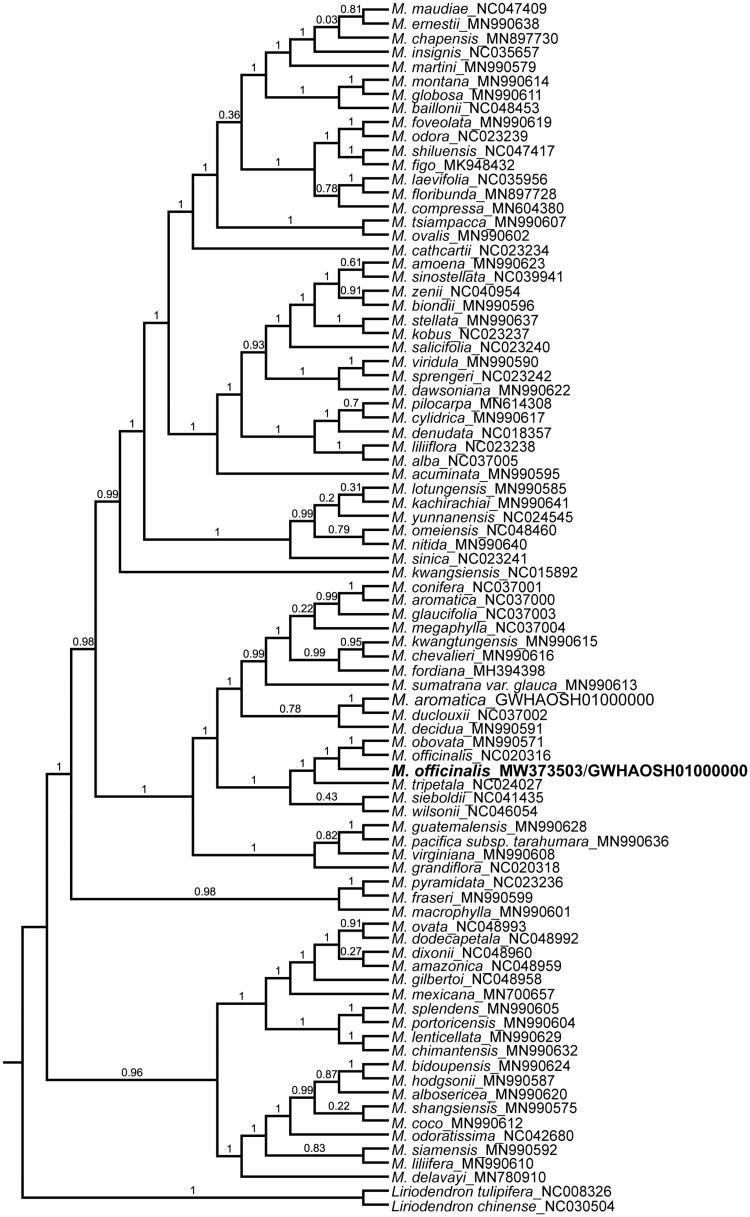
Phylogenetic tree inferred by Maximum Likelihood (ML) method based on 86 representative species. *Liriodendron tulipifera* and *Liriodendron chinese* were used as outgroup. A total of 1000 bootstrap replicates were computed and the bootstrap support values are shown at the branches. Accession numbers were shown in Figure 1.

## Data Availability

The genome sequence data of *M. officinalis* that support the findings of this study are openly available in GenBank of NCBI at (https://www.ncbi.nlm.nih.gov/) under the accession no. MW373503. The associated BioProject, SRA, and Bio-Sample numbers are PRJNA685116, SRR13251560, and SAMN17076828, respectively. Meanwhile, the complete chloroplast genome sequence of *M. officinalis* is also deposited in the Genome Warehouse (https://bigd.big.ac.cn/search/?dbId=gwh&q=GWHAOTT01000000) database under the accession number: GWHAOTT01000000. The raw sequencing data is deposited in the Genome Sequence Archive database (https://bigd.big.ac.cn/gsa/browse/CRA003363/CRR195297) under the accession number: CRR195297.
